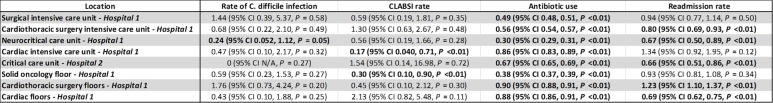# 22 Antibiotic-Related Allergy in Hospitalized Children in a Korean Tertiary Hospital: A High Frequency of Inappropriate Management

**DOI:** 10.1017/ash.2026.10469

**Published:** 2026-06-23

**Authors:** Jon Johannesson, Erin Gettler, Nitin Mehdiratta, Heather Pena, Rebekah Moehring, Jessica Seidelman

**Affiliations:** 1 Duke University Hospital; 2 Duke University Medical Center; 3 Duke University

## Abstract

**Background:** Blood culture (BCx) algorithms can safely and effectively reduce unnecessary BCxs. We compared the effect of implementing a BCx algorithm across multiple inpatient units within our hospital system, focusing on blood culture event (BCE) rates and secondary adverse events. **Method:** The same BCx algorithm (Figure 1) was implemented in multiple inpatient units across three hospitals in our health system (Figure 2). BCE rates were compared between pre- and post-intervention periods with interrupted time series (ITS) analyses. Differences in secondary outcomes, including incidence of central line-associated bloodstream infections (CLABSI) and Clostridioides difficile infection (CDI), were compared using χ tests. **Result:** The rate of BCE decreased after implementation of the BCx algorithm across all units (Figure 3). An immediate decrease in BCE rate was noted in most units upon implementation of the algorithm, except in the cardiac ICU, cardiac floors and neurocritical care unit. No unit demonstrated a significant post-intervention decrease in the BCE rate, with the surgical ICU demonstrating a significant regression to the mean with time. Antibiotic use decreased across all analyzed units (Figure 4). Readmission rates decreased significantly on the cardiac floor, neurocritical care unit and cardiothoracic ICU in hospital 1 and also in the critical care unit in hospital 2, while it increased significantly in the cardiothoracic stepdown unit. CLABSI rates decreased significantly in the cardiac ICU and the solid oncology floor, while CDI rates decreased significantly in the neurocritical care unit. No significant increase was seen in CLABSI and CDI rates or antibiotic use. **Conclusion:** This study demonstrated that the BCx algorithm was effective in decreasing BCEs across all units and hospitals without any clear harms. However, the effect of BCx algorithms is partially dependent on the nature of the unit where it is implemented. The decrease in BCEs was limited to the time period directly after implementation of the algorithm. No units demonstrated ongoing decreases in BCE rates, with the surgical ICU in particular demonstrating a steady increase of BCE rates as time progressed, although rates of BCE remained below pre-intervention levels. Consistent decreases were seen in antibiotic use post-implementation, although causality between the two cannot be determined. Further research is warranted on how to implement a more durable change and on the use of such algorithms in more specific populations, including immunocompromised hosts.

**Figure:**
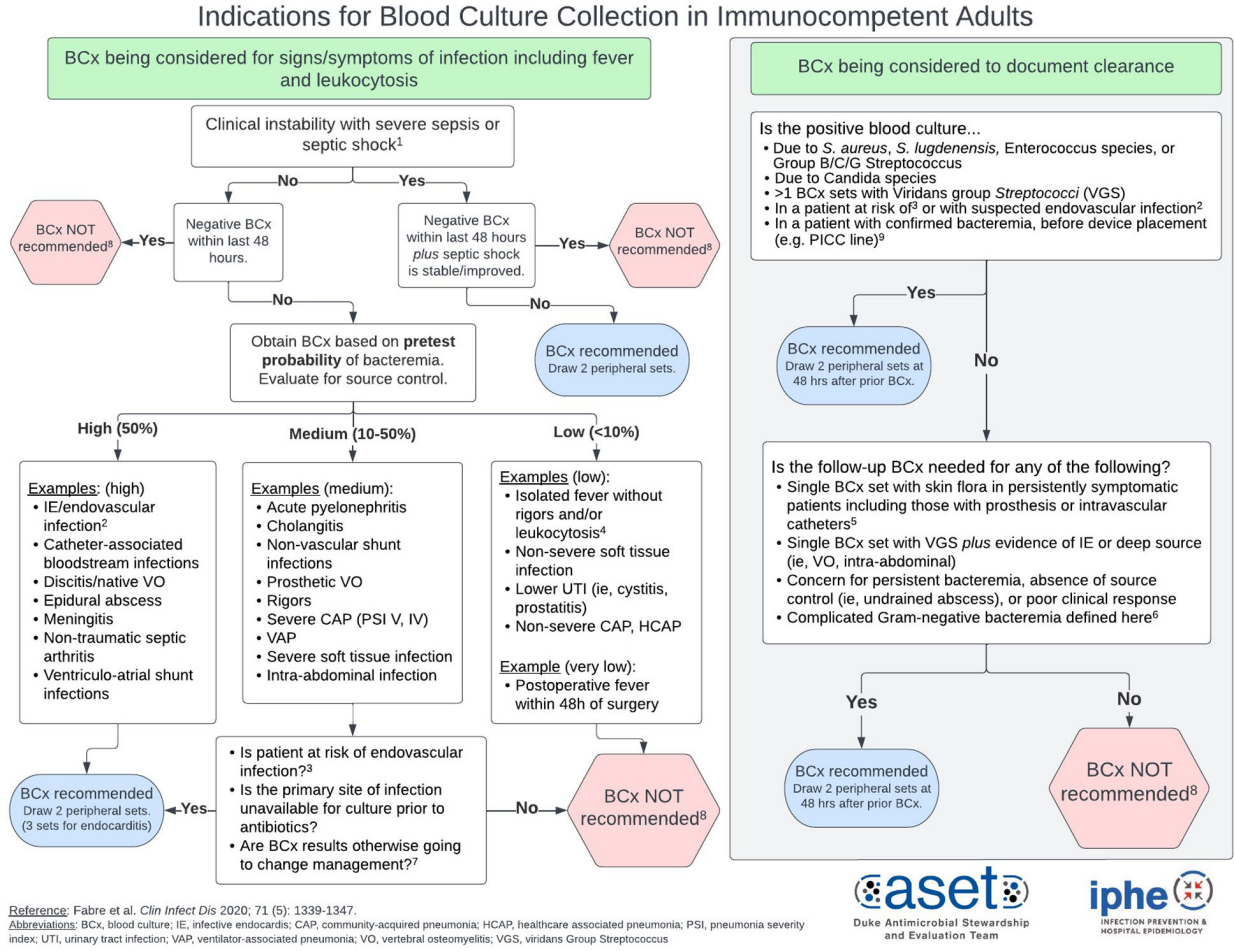

**Figure:**
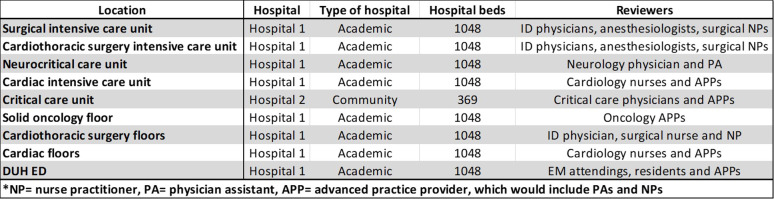

**Figure:**
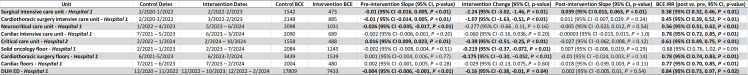

**Figure:**